# Patient Characteristics and the Extent to Which Clinicians Involve Patients in Decision Making: Secondary Analyses of Pooled Data

**DOI:** 10.1177/0272989X241231721

**Published:** 2024-03-04

**Authors:** Sascha M. Keij, Megan E. Branda, Victor M. Montori, Juan P. Brito, Marleen Kunneman, Arwen H. Pieterse

**Affiliations:** Medical Decision Making, Department of Biomedical Data Sciences, Leiden University Medical Center, The Netherlands; Knowledge and Evaluation Research Unit, Mayo Clinic, Rochester MN, USA; Knowledge and Evaluation Research Unit, Mayo Clinic, Rochester MN, USA; Knowledge and Evaluation Research Unit, Mayo Clinic, Rochester MN, USA; Medical Decision Making, Department of Biomedical Data Sciences, Leiden University Medical Center, The Netherlands; Knowledge and Evaluation Research Unit, Mayo Clinic, Rochester MN, USA; Medical Decision Making, Department of Biomedical Data Sciences, Leiden University Medical Center, The Netherlands

**Keywords:** shared decision making, patient involvement, patient characteristics, pooled analyses

## Abstract

**Background:**

The occurrence of shared decision making (SDM) in daily practice remains limited. Various patient characteristics have been suggested to potentially influence the extent to which clinicians involve patients in SDM.

**Objective:**

To assess associations between patient characteristics and the extent to which clinicians involve patients in SDM.

**Methods:**

We conducted a secondary analysis of data pooled from 10 studies comparing the care of adult patients with (intervention) or without (control) a within-encounter SDM conversation tool. We included studies with audio(-visual) recordings of clinical encounters in which decisions about starting or reconsidering treatment were discussed.

**Main Measures:**

In the original studies, the Observing Patient Involvement in Decision Making 12-items (OPTION^12 item^) scale was used to code the extent to which clinicians involved patients in SDM in clinical encounters. We conducted multivariable analyses with patient characteristics (age, gender, race, education, marital status, number of daily medications, general health status, health literacy) as independent variables and OPTION^12^ as a dependent variable.

**Results:**

We included data from 1,614 patients. The between-arm difference in OPTION^12^ scores was 7.7 of 100 points (*P* < 0.001). We found no association between any patient characteristics and the OPTION^12^ score except for education level (*p* = 0.030), an association that was very small (2.8 points between the least and most educated), contributed mostly by, and only significant in, control arms (6.5 points). Subanalyses of a stroke prevention trial showed a positive association between age and OPTION^12^ score (*P* = 0.033).

**Conclusions:**

Most characteristics showed no association with the extent to which clinicians involved patients in SDM. Without an SDM conversation tool, clinicians devoted more efforts to involve patients with higher education, a difference not observed when the tool was used.

**Highlights:**

- Most sociodemographic patient characteristics show no association with the extent to which clinicians involve patients in shared decision making.
- Clinicians devoted less effort to involve patients with lower education, a difference that was not observed when a shared decision-making conversation tool was used.
- SDM conversation tools can be useful for clinicians to better involve patients and ensure patients get involved equally regardless of educational background.

## Introduction

In the past decades, there has been a growing focus on incorporating patients’ values, preferences, and goals in medical decision making.^1,2^ To bring these preferences and values to medical decisions, clinicians are expected to engage patients in shared decision making (SDM). However, the occurrence of SDM in daily practice remains limited.^3–5^

SDM requires collaboration between clinicians and patients.^6–9^ It can be difficult for patients to achieve what it requires from them to become truly involved in decision making. This may be more difficult for some patients than for others.^10–12^ Clinicians and patients may believe that certain characteristics affect a patient’s ability or willingness to be involved.^12^ For example, older patients or those with lower educational background or limited health literacy may struggle with aspects such as information processing and communication. Patients from minority ethnic backgrounds may expect a paternalistic decision-making style, and experience difficulties in information exchange. Health-related factors, such as having severe symptoms or competing medical problems, may affect choice perception or limit the ability to explore options.^12^ Clinicians may be less likely to involve patients they believe would not want to or are less able to participate in SDM.^13,14^ Clinicians may prejudicially assume that patients with certain characteristics are less willing or less able to participate in SDM. Thus, patient characteristics may act as barriers or facilitators to SDM as they relate to clinicians’ beliefs and assumptions about the worthiness of doing the intellectual, emotional, and practical work to involve patients in SDM. It remains unclear whether particular patient characteristics are associated with clinicians’ efforts to involve patients in SDM.^15^

As a first step to identify potential associations, we conducted a scoping review. We found more than 70 patient-related characteristics about which studies had explored their association with SDM.^15^ Most often, these studies explored the association with patient sociodemographic characteristics, such as age and educational background. Studies produced inconsistent results. Studies were included that measured SDM occurrence regardless of which measurement instrument was used (ranging from 1-item self-developed questions to using multiple often-used SDM instruments) or from which perspective it was measured (i.e., the patient, the clinician, and/or an independent observer), complicating the interpretability of the results.

In this study, we focused on a third-party observer’s perspective. Although all perspectives are relevant in measuring SDM, an observer’s perspective allows for the most objective assessment of the extent to which clinicians made discernible efforts to involve patients in SDM. We conducted secondary pooled analyses of completed trials of using an SDM intervention versus usual care to assess associations between patient characteristics and the extent to which clinicians involved patients in SDM, as measured from an observer’s perspective.

## Methods

### Design

We conducted a secondary analysis of pooled data. We used data from studies evaluating the efficacy of interventions to promote SDM led by investigators at the Knowledge and Evaluation Research Unit (KER unit, Mayo Clinic, USA). Studies were conducted between 2007 and 2019. Analyses were conducted between 2022 and 2023. We aimed to assess whether patient sociodemographic characteristics are associated with more patient involvement in health care decisions from an observer perspective.

### Study Inclusion

We included studies 1) enrolling adult patients and their clinicians, 2) comparing care outcomes between usual care encounters with and without a within-encounter SDM conversation tool, 3) in which the encounter in which a medical decision was discussed was recorded (audio-visual or audio only), and 4) the recordings were coded using the Observing Patient Involvement in Decision Making 12-item (OPTION^12 item^) scale.^16^

### Study Characteristics

We included data from 10 different studies (Table 1). All studies were conducted in various outpatient and inpatient settings in suburban and rural health care facilities in the upper Midwest, Alabama, and Mississippi, USA. Studies focused on different disease states (i.e., chest pain, diabetes, Graves’ disease, osteoporosis, depression, atrial fibrillation, or cardiovascular primary prevention). All studies included patients who faced actual decisions about starting or reconsidering treatment during a clinical encounter. Prior to the encounter, patients were asked if they wanted to participate in the study. For 9 studies, patients who indicated they wanted to participate and who provided informed consent were randomly assigned to an encounter with care as usual or in which a within-encounter SDM conversation tool was used. One study had a pre-post implementation design. Two types of conversation tools were used. Risk calculators were used for decisions that required communication about the individual patient’s risks relating to the choice of a risk-reducing treatment. Issue cards were used to compare the characteristics of treatment options based on their importance to the patients and to aid in the selection of an appropriate treatment option. Prior to using the conversation tool, clinicians (physicians, nurse practitioners, physician assistants, or registered nurses) had received a short training on how to use the conversation tool during the encounter. Encounters were recorded if both the patient and clinician provided consent.

**Table 1:** Characteristics of Included Studies

Trial Name	Year	Disease State	Patients (*n*)	Clinicians (*n*)	Clinical Context	Study Site	Conversation Tool Type	Details of Choice
Chest Pain^17^	2010–2011	Chest pain	204	51	Emergency department visit for chest pain	Suburban	Risk calculator	Admission for observation or discharge with follow-up
DAD^18^	2010–2011	Diabetes/cardiovascular primary prevention	104	41	Primary care clinic visit for diabetic management	Suburban and rural	Issue cards/risk calculator	Multiple drug options for diabetes: oral v. injectable medications/statin or not
Diabetes^19^	2007–2008	Diabetes	85	40	Primary care clinic visit for diabetic management	Suburban and rural	Issue cards	Multiple drug options for diabetes: oral v. injectable medications
Graves^20^	2013–2015	Graves’ disease	68	16	Outpatient discussion within the endocrinology department at Mayo clinic	Suburban	Issue cards	Treatment choice: antithyroid drugs, radioiodine ablation, surgery
Osteo I^21^	2007–2008	Osteoporosis	100	61	Primary care clinic visit for osteoporosis prevention	Suburban	Risk calculator	Bisophosphonate or not
Osteo II^22^	2009–2010	Osteoporosis	79	40	Primary care clinic visit for osteoporosis prevention	Suburban	Risk calculator	Bisophosphonate or not
iADAPT^23^	2011–2013	Depression	297	117	Primary care clinic for depression management	Suburban and rural	Issue cards	Multiple drug options for depression
SDM4Afib^24^	2017–2019	Atrial fibrillation	922	151	Cardiology outpatient and primary care	Suburban and rural	Risk calculator/issue cards	Whether or not to use an anticoagulant and which one
Statin^25^	2005	Cardiovascular primary prevention	93	21	Diabetes clinic visit for prevention of cardiovascular disease	Suburban	Risk calculator	Statin or not
TRICEP^26^	2011–2013	Diabetes	350	99	Primary care clinic visit for diabetic management	Suburban and rural	Issue cards	Multiple drug options for diabetes: oral v. injectable medications

### Study Instruments

All data were retrieved from a data set from the KER unit.

#### Patient involvement in SDM

In all studies, and as part of data collection in the original studies, patient involvement in decision making was measured with the OPTION^12^ scale.^16^ The OPTION^12^ is an observer-based coding scale that measures the extent to which clinicians involve patients in decision making. The scale consists of 12 items scored from 0 (minimal effort to involve patients) to 4 (maximum effort to involve patients). Trained pairs of investigators reviewed and coded recordings with good interobserver agreement. The total score is the sum of all items, converted to a 0- to 100-point scale, where higher values indicate more patient engagement in the conversation by the clinician.

#### Patient characteristics

In all studies, patient demographics were collected through chart reviews and self-report questionnaires (i.e., gender assigned at birth, age, race, education, and marital status). Questionnaires were filled in by patients directly before or after the encounter. We used data from the “confidence in filling out medical forms without assistance” question as a screening item for health literacy.^27^ Perceived general health status was measured with a single item, “In general, would you say your health is . . .,” with a 5-point scale ranging from *poor* to *excellent*.^28^ We included the total number of medications (either collected from chart review or collected through the patient questionnaire) as an indirect measure of treatment complexity.

### Statistical Analysis

Patient characteristics are described with means and standard deviations for continuous outcomes and counts and frequencies for categorical variables. After examining the distribution of OPTION^12^ scores, we assumed normality. We conducted univariate generalized linear model analyses with a random effect of study for each characteristic of interest (age, gender, race, education, marital status, medication count, general health status, and health literacy). A multivariable model was used to assess the association of OPTION^12^ with patient characteristics (i.e., age, gender, race, education, marital status, medication count) that were collected in all studies. Interaction effects were tested using the likelihood ratio test for all 2-way combinations of adjusted characteristics. We further ran separate models for the usual care and conversation tool arms. As approximately half of the participants originated from a single study (SDM4AFIB), we conducted a post hoc sensitivity analysis in which this trial was excluded.

The patient characteristics general health status and health literacy were not collected consistently across studies. To assess potential associations between these characteristics and OPTION^12^ scores, we ran an additional model for the study (SDM4AFIB) that collected both of these characteristics. All statistical analyses were performed using SAS v. 9.4 (SAS, Cary, NC).

## Results

We included data from a total of 1,614 patients (Table 2). Patient characteristics per study can be found in Appendix A.

**Table 2:** Patient Characteristics (*N* = 1,614)

Characteristic	*n* (%)^a^
Arm	
Shared decision-making conversation tool	849 (52.6)
Age, y	
$\bar x$ (*s*)	64.3 (14.1)
<55	357 (22.1)
55–64	380 (23.5)
65–74	477 (29.6)
75+	400 (24.8)
Gender	
Female	782 (48.5)
Race	
White/Caucasian	1,383 (88.9)
Black, Indigenous, People of Color (BIPOC)	173 (11.1)
Asian	19 (1.2)
Black/African American	124 (8.0)
Other	21 (1.3)
American Indian/Alaskan Native	9 (0.6)
Missing	58
Education	
< High school	229 (16.0)
High school grade/GED	456 (31.9)
Some college/vocational school	462 (32.3)
College/postgraduate degree	284 (19.8)
Missing	183
Married	
Married/marriage-like relationship	915 (65.1)
Single/divorced/widowed	491 (34.9)
Missing	208
General health	
Excellent/very good	307 (33.7)
Good	408 (44.7)
Fair/poor	197 (21.6)
Missing	702
Total number of medications	
$\bar x$ (*s*)	7.5 (4.48)
0–4	310 (24.5)
5–9	598 (47.3)
10–14	357 (28.2)
Missing	349
Health literacy: confidence in filling forms	
Not at all/little bit	77 (8.6)
Somewhat	119 (13.4)
Quite a bit	268 (30.1)
Extremely	427 (47.9)
Missing	723
Name of study	
Chest Pain	201 (12.5)
DAD	41 (2.5)
Diabetes	51 (3.2)
Graves	55 (3.4)
IADAPT	96 (5.9)
Osteo I	70 (4.3)
Osteo II	38 (2.4)
SDM4Afib	830 (51.4)
Statin Choice	91 (5.6)
TRICEP	141 (8.7)

a Percentage calculated based on actual *n* not including missing values.

### Univariable Analyses

No patient characteristics were significantly associated with OPTION^12^ scores (Table 3). Patients who used the conversation tool with their clinicians had significantly higher OPTION^12^ scores compared with those in the usual care group (*P* < 0.001).

**Table 3:** Univariable Model Findings (*N* = 1,614)

Patient Characteristic	OPTION^12^ Score Least Square Mean (95% CI)	*P* Value
Age, y		0.105
<55	33.3 (25.1, 41.5)	
55–64	32.2 (24.1, 40.3)	
65–74	32.4 (24.3, 40.4)	
≥75	33.3 (25.1, 41.5)	
Gender		0.23
Female	33.4 (23.7, 43.1)	
Male	32.3 (22.5, 42.0)	
Race		0.059
White	33.17 (23.4, 42.9)	
Black, Indigenous, People of Color	30.03 (19.8, 40.2)	
Education		0.094
<High school	31.2 (23.0, 39.5)	
High school/GED	33.1 (25.0, 41.2)	
Some college/vocational	32.0 (24.0, 40.1)	
College/postgraduate	31.2 (23.0, 39.5)	
Marital status		0.34
Married/marriage-like relationship	32.0 (23.8, 40.3)	
Other (single, divorced, separated, widowed)	32.8 (24.5, 41.1)	
Total number of medications		0.45
0–4	33.2 (24.9, 41.6)	
5–9	31.9 (23.55, 40.3)	
≥10	32.9 (24.6, 41.2)	
Intervention arm		<0.001
Usual care	29.0 (19.6, 38.4)	
SDM conversation tool	36.7 (27.3, 46.2)	
General health^a^		0.62
Excellent/very good	27.5 (13.5, 41.5)	
Good	26.5 (12.6, 40.4)	
Fair/poor	26.7 (12.5, 40.8)	
Health literacy^b^—confidence filling out medical forms		0.39
Not at all/a little bit	24.2 (21.7, 46.6)	
Somewhat	37.6 (25.4, 49.8)	
Quite a bit	35.9 (24.0, 47.8)	
Extremely	36.7 (24.9, 48.5)	

CI, confidence interval; SDM, shared decision making.

a Missing for all studies except SDM4Afib (*N* = 786) and TRICEP (*N* = 126).

b Missing for all studies except SDM4Afib (*N* = 800) and IADAPT (*N* = 91).

### Multivariable Analyses

In the multivariable analyses, educational background was significantly associated with OPTION^12^ scores (*p*=0.030). Encounters with higher educated patients generally had slightly higher OPTION^12^ scores. We found a significant interaction between age and arm (Appendix B). The interaction did not appear clinically meaningful and was therefore not included in the final model (Table 4). A sensitivity analysis excluding the largest trial (SDM4AFIB) reduced the precision of the estimates but did not affect the magnitude of association between OPTION^12^ score and each patient characteristic evaluated (Appendix C).

**Table 4:** Multivariable Model Findings (*n* = 1,104)

Patient Characteristic	OPTION^12^ Score Least Square Mean (95% CI)	*P* Value
Age, y		0.29
<55	31.4 (23.4, 39.4)	
55–64	31.2 (23.3, 39.1)	
65–74	31.4 (23.5, 39.3)	
≥75	33.1 (25.1, 41.1)	
Gender		0.156
Female	32.5 (22.9, 42.1)	
Male	31.1 (21.4, 40.8)	
Race		0.112
White	33.0 (23.5, 42.5)	
Other	30.6 (20.6, 40.5)	
Education		0.030
<High school	30.1 (22.0, 38.1)	
High school/GED	32.3 (24.3, 40.2)	
Some college/vocational	30.9 (23.0, 38.8)	
College/postgraduate	33.9 (25.9, 41.9)	
Marital status		0.095
Married/marriage-like relationship	31.0 (22.1, 39.8)	
Other (single, divorced, separated, widowed)	32.6 (23.7, 41.5)	
Total number of medications		0.46
0–4	32.5 (24.3, 40.6)	
5–9	31.8 (22.9, 39.2)	
≥10	31.0 (22.9, 39.2)	
Intervention arm		<0.001
Usual care	27.9 (19.1, 36.8)	
SDM conversation tool	35.6 (26.8, 44.5)	

CI, confidence interval; SDM, shared decision making.

Separate analyses for the usual care and SDM conversation tool arm showed a significant association between education and OPTION^12^ scores in the usual care arm; patients with the lowest educational background had OPTION^12^ scores of 6.5 points lower than patients with the highest educational background (*p* = .017). There was no association between educational background and OPTION^12^ scores in the SDM conversation tool arm (Table 5). In the usual care arm, patients between 55 and 64 y old had OPTION^12^ scores 5.1 of 100 points lower than patients aged 75 y or older (*p* = 0.051).

**Table 5:** Multivariable Model Findings per Arm (*n* = 1,104)

	Usual Care (*n* = 527)		SDM Conversation Tool (*n* = 577)	
Patient Characteristic	OPTION^12^ Score Least Square Mean (95% CI)	*P* Value	OPTION^12^ Score Least Square Mean (95% CI)	*P* Value
Age, y		0.051		0.46
<55	24.8 (17.1, 32.4)		38.7 (28.8, 48.6)	
55–64	21.2 (13.9, 28.5)		40.3 (30.5, 50.1)	
65–74	23.9 (16.5, 31.3)		38.1 (28.3, 47.9)	
≥75	26.3 (18.7, 33.8)		39.5 (29.6, 49.4)	
Gender		0.167		0.31
Female	25.0 (16.3, 33.7)		39.8 (27.9, 51.7)	
Male	23.1 (14.2, 31.9)		38.5 (26.5, 50.5)	
Race		0.32		0.157
White	25.1 (16.0, 34.1)		40.6 (27.9, 53.3)	
Other	23.0 (12.8, 33.2)		37.7 (27.9, 53.3)	
Education		0.017		0.86
<High school	21.0 (13.5, 28.6)		38.7 (28.7, 48.8)	
High school/GED	24.5 (17.3, 31.8)		39.0 (29.1, 48.9)	
Some College/vocational	23.1 (15.9, 30.2)		38.8 (29.0, 48.7)	
College/postgraduate	27.5 (20.0, 35.0)		40.0 (30.0, 50.0)	
Marital status		0.107		0.35
Married/marriage-like relationship	22.9 (14.9, 30.9)		38.6 (27.6, 49.6)	
Other (single, divorced, separated, widowed)	25.2 (17.0, 33.3)		39.7 (28.6, 50.7)	
Total number of medications		0.64		0.70
0–4	24.7 (17.1, 32.3)		39.9 (29.5, 50.2)	
5–9	24.2 (16.8, 31.5)		38.7 (28.5, 49.0)	
≥10	23.2 (15.7, 30.7)		38.9 (28.5, 49.2)	

CI, confidence interval; SDM, shared decision making.

### Multivariable Analyses for General Health Status and Health Literacy

General health status and health literacy showed no significant associations with OPTION^12^ scores in the model with only data from the SDM4Afib trial included (Table 6). There was a significant association between age and OPTION^12^ scores (*p* = 0.033). Encounters with patients who were 75 y or older had OPTION^12^ scores that were 3.3 points higher than encounters with patients younger than 55 y. There were no significant interaction effects.

**Table 6:** Multivariable Model Findings for General Health Status and Health Literacy (SDM4Afib Trial Only; *N* = 756)

Patient Characteristic	OPTION^12^ Score Least Square Mean (95% CI)	*P* Value
Age, y		
<55	29.8 (26.2, 33.4)	0.033
55–64	30.2 (28.1, 32.7)	
65–74	30.8 (29.1, 32.4)	
≥75	33.1 (31.6, 34.6)	
Gender		0.077
Female	31.8 (29.9, 33.6)	
Male	30.2 (28.8, 31.6)	
Education		
<High school	29.1 (27.2, 31.0)	0.060
High school/GED	32.1 (30.3, 33.8)	
Some college/vocational	29.7 (27.6, 31.9)	
College/Postgraduate	33.0 (30.6, 35.4)	
Intervention arm		
Usual care	28.7 (27.1, 30.3)	<0.001
SDM conversation tool	33.3 (31.7, 34.9)	
General health		
Fair/poor	31.2 (29.3, 33.1)	0.77
Good	30.6 (28.9, 32.2)	
Excellent/very good	31.2 (29.2, 33.1)	
Health literacy confidence		0.161
Not at all	29.0 (25.9, 32.2)	
Somewhat	33.1 (30.6, 35.6)	
Quite a bit	30.6 (28.8, 32.4)	
Extremely	31.2 (29.7, 32.6)	

CI, confidence interval; SDM, shared decision making.

## Discussion

The purpose of this study was to identify potential associations between patient characteristics and the extent to which clinicians involve patients in SDM. We combined data from 10 clinical studies, focused on treatment decisions in primary and specialty care. In the overall pooled analyses, education was the only characteristic with a significant association. In line with our expectations, clinicians showed the lowest level of patient-involving behaviors with patients with the lowest educational background and the highest level with patients with the highest educational background. Additional analyses showed that education was associated with patient involvement only in the usual care arm and not in the SDM conversation tool arm. This shows that SDM conversation tools may thus be an effective way of overcoming the negative influence educational background may have on the extent to which clinicians involve patients in decision making. In previous research assessing the association between education and the occurrence of SDM about treatment, most studies found no associations.^15^ There were 2 studies that assessed SDM with the OPTION^12^, both also showing no significant associations. One of these studies had a very small sample size (*N* = 19),^29^ limiting the ability to detect potential associations. The other study (*N* = 80) looked at cancer treatment decisions and showed an average OPTION^12^ score ($\bar x$ = 15.7, *s* = 9.0) that was much lower than in our sample, indicating that clinicians generally showed few behaviors to involve patients,^30^ regardless of education level.

In the overall analyses, there was no significant association between age and the extent to which clinicians involved patients, in line with most previous studies.^3,15^ In the subanalyses of the SDM4Afib trial (*N* = 756), age showed a significant effect, namely, clinicians demonstrated more efforts to involve older (≥75 y) compared with younger (<55 y) patients. This particular trial was focused on decisions about anticoagulation treatment to reduce the risk of stroke. The risk of stroke increases with age,^31^ but so do the risks of anticoagulation treatment.^32^ In older patients, the best way forward may thus be less clear to clinicians, which may have made clinicians more likely to involve patients in the decision.^13^

None of the other patient characteristics were associated with the extent to which clinicians tried to involve patients. Clinicians thus appeared to involve patients equally, regardless of any of those characteristics. This is in line with previous research, which demonstrated a lack of clear associations between patient characteristics and SDM occurrence.^15^ It is also possible that some patient characteristics may have had opposing effects, canceling potential associations. For example, SDM may be more relevant in patients with multiple medical conditions requiring treatment, due to the complexity of combining treatments, and there may be less evidence to favor one treatment option over another. On the other hand, involvement can also be more difficult as there are more factors to consider, such as how different medications interact with each other. Comorbidities thus have the potential to make clinicians either more or less likely to try to involve patients in decision making. To fully assess such potential associations, we would need to adopt a study design that allows for disentangling such potentially opposing effects.

In the analyses, we included patient characteristics that had been consistently assessed across studies, which related only to sociodemographic and health-related characteristics. However, other characteristics, such as emotional distress or the patient-clinician relationship, may also play a role in the extent to which clinicians aim to involve patients.^12^ For instance, clinicians may be less likely to involve patients who are highly anxious, as they may believe SDM is inappropriate in such situations or they may perceive the patients as less capable of becoming involved in that state. On the other hand, clinicians may be more likely to involve patients whom they perceive to trust them.^33^

### Implications

Clinicians appear to be able to involve patients in SDM to the same extent regardless of any of the assessed patient characteristics, with the exception of educational background. Conversation tools can be useful for clinicians to better involve patients and can help ensure that patients get involved equally regardless of educational background. Clinicians should, when possible and appropriate, try to involve patients in decision making regardless of patients’ characteristics.

### Strengths and Limitations

In this study, we combined data from multiple studies that assessed SDM using a frequently used observer-based SDM instrument. We were able to include a range of patient characteristics and to test potential associations. Our findings are limited by the fact that the study populations varied across time, disease state, and setting. Our decision to include a diverse set of studies may have reduced our ability to detect associations, as these associations interact with study characteristics such as clinical situation, trial intervention, and health care setting. These potential interactions may have obscured associations or confounded observed ones. Furthermore, our study has some limitations with regard to the included variables. First, we were not able to include other characteristics that may have affected the extent to which clinicians aimed to involve patients, such as emotional distress, quality of life, or preferences for decision making. This was either because such aspects had not been measured across studies or because variables had been measured after the encounter, making it possible that patients’ answers were affected by the extent to which SDM had taken place. This limited the ability to assess whether such characteristics may have affected clinician behavior or the other way around. Second, for the scope of this project, we looked only at patient characteristics, whereas (the interaction with) clinician characteristics could also have been relevant to explore. Third, health literacy had been assessed with a single-item screening question. This item has been previously shown to perform moderately well on identifying patients with inadequate and marginal health literacy, although more extensive and accurate tools are available.^34^

## Conclusions

Most patient characteristics showed no association with the extent to which clinicians involved patients in decision making and do not appear to be predictive of whether SDM is possible. Clinicians should thus not refrain from trying to involve patients, regardless of any such patient characteristics. In the overall analyses, education was the only characteristic associated with the extent to which clinicians involved patients in decision making. The use of an SDM conversation tool appeared to counter this negative effect and may thus be especially useful in encounters with patients with low education levels. The SDM conversation tool can thus be an effective way to involve patients to an equal extent regardless of educational background. Other patient-related characteristics that we did not include in this study may be related to the extent to which clinicians involve patients in SDM. Further research should focus on disentangling if and how psychological or relationship-related characteristics, such as anxiety or trust, are related to the extent to which clinicians try to involve patients in decision making.

## Supplemental Material

sj-docx-1-mdm-10.1177_0272989X241231721 – Supplemental material for Patient Characteristics and the Extent to Which Clinicians Involve Patients in Decision Making: Secondary Analyses of Pooled Data Supplemental material, sj-docx-1-mdm-10.1177_0272989X241231721 for Patient Characteristics and the Extent to Which Clinicians Involve Patients in Decision Making: Secondary Analyses of Pooled Data by Sascha M. Keij, Megan E. Branda, Victor M. Montori, Juan P. Brito, Marleen Kunneman and Arwen H. Pieterse in Medical Decision Making

sj-docx-2-mdm-10.1177_0272989X241231721 – Supplemental material for Patient Characteristics and the Extent to Which Clinicians Involve Patients in Decision Making: Secondary Analyses of Pooled Data Supplemental material, sj-docx-2-mdm-10.1177_0272989X241231721 for Patient Characteristics and the Extent to Which Clinicians Involve Patients in Decision Making: Secondary Analyses of Pooled Data by Sascha M. Keij, Megan E. Branda, Victor M. Montori, Juan P. Brito, Marleen Kunneman and Arwen H. Pieterse in Medical Decision Making

sj-docx-3-mdm-10.1177_0272989X241231721 – Supplemental material for Patient Characteristics and the Extent to Which Clinicians Involve Patients in Decision Making: Secondary Analyses of Pooled Data Supplemental material, sj-docx-3-mdm-10.1177_0272989X241231721 for Patient Characteristics and the Extent to Which Clinicians Involve Patients in Decision Making: Secondary Analyses of Pooled Data by Sascha M. Keij, Megan E. Branda, Victor M. Montori, Juan P. Brito, Marleen Kunneman and Arwen H. Pieterse in Medical Decision Making
