# Supplementary material for: Patient Characteristics and the Extent to Which Clinicians Involve Patients in Decision Making: Secondary Analyses of Pooled Data
Source: Med Decis Making. 2024 Mar 4;44(3):346–56. doi: 10.1177/0272989X241231721 (PMC10988989; doi:10.1177/0272989X241231721)
Supplement: sj-docx-1-mdm-10.1177_0272989X241231721 – Supplemental material for Patient Characteristics and the Extent to Which Clinicians Involve Patients in Decision Making: Secondary Analyses of Pooled Data [file sj-docx-1-mdm-10.1177_0272989X241231721.docx]

**Appendix A.** Patient characteristics and OPTION^12^ scores

Table 1. Patient characteristics.

|  | Name of Study | | | | | | | | | |  | |
| --- | --- | --- | --- | --- | --- | --- | --- | --- | --- | --- | --- | --- |
|  |  | | | | | | | | | |  | |
|  | Chest Pain (N=201) | DAD (N=41) | Diabetes (N=51) | Osteo I (N=70) | Osteo II (N=38) | Statin Choice (N=91) | Graves Disease (N=55) | IADAPT (N=96) | TRICEP (N=141) | SDM4Afib (N=830) | Total (N=1614) | *P*-value |
| **Arm**, n (%) |  |  |  |  |  |  |  |  |  |  |  | 0.02^1^ |
| Control | 100 (49.8) | 19 (46.3) | 21 (41.2) | 33 (47.1) | 25 (65.8) | 42 (46.2) | 27 (49.1) | 39 (40.6) | 48 (34.0) | 411 (49.5) | 765 (47.4) |  |
| DA | 101 (50.2) | 22 (53.7) | 30 (58.8) | 37 (52.9) | 13 (34.2) | 49 (53.8) | 28 (50.9) | 57 (59.4) | 93 (66.0) | 419 (50.5) | 849 (52.6) |  |
|  |  |  |  |  |  |  |  |  |  |  |  |  |
| **Age** |  |  |  |  |  |  |  |  |  |  |  | <.0001^2^ |
| N | 201 | 41 | 51 | 70 | 38 | 91 | 55 | 96 | 141 | 830 | 1614 |  |
| Mean (SD) | 54.6 (11.82) | 57.8 (11.23) | 62.9 (11.61) | 66.5 (8.77) | 65.8 (10.00) | 65.6 (10.25) | 41.8 (14.08) | 45.5 (15.47) | 60.0 (10.61) | 71.0 (10.43) | 64.3 (14.10) |  |
| Median | 54.0 | 58.0 | 64.0 | 68.0 | 64.0 | 67.0 | 39.0 | 47.5 | 61.0 | 71.0 | 66.0 |  |
| Range | 27.0, 87.0 | 26.0, 80.0 | 39.0, 89.0 | 50.0, 84.0 | 50.0, 86.0 | 37.0, 83.0 | 18.0, 76.0 | 19.0, 79.0 | 23.0, 85.0 | 31.0, 98.0 | 18.0, 98.0 |  |
|  |  |  |  |  |  |  |  |  |  |  |  |  |
| **Age**, n (%) |  |  |  |  |  |  |  |  |  |  |  | <.0001^1^ |
| <55 | 110 (54.7) | 15 (36.6) | 12 (23.5) | 7 (10.0) | 3 (7.9) | 13 (14.3) | 43 (78.2) | 61 (63.5) | 43 (30.5) | 50 (6.0) | 357 (22.1) |  |
| 55-64 | 53 (26.4) | 16 (39.0) | 17 (33.3) | 19 (27.1) | 16 (42.1) | 23 (25.3) | 10 (18.2) | 26 (27.1) | 49 (34.8) | 151 (18.2) | 380 (23.5) |  |
| 65-74 | 25 (12.4) | 7 (17.1) | 15 (29.4) | 30 (42.9) | 12 (31.6) | 35 (38.5) | 1 (1.8) | 8 (8.3) | 38 (27.0) | 306 (36.9) | 477 (29.6) |  |
| 75+ | 13 (6.5) | 3 (7.3) | 7 (13.7) | 14 (20.0) | 7 (18.4) | 20 (22.0) | 1 (1.8) | 1 (1.0) | 11 (7.8) | 323 (38.9) | 400 (24.8) |  |
|  |  |  |  |  |  |  |  |  |  |  |  |  |
| **Gender**, n (%) |  |  |  |  |  |  |  |  |  |  |  | <.0001^1^ |
| Female | 118 (58.7) | 12 (29.3) | 22 (43.1) | 70 (100.0) | 38 (100.0) | 41 (45.1) | 42 (76.4) | 62 (64.6) | 58 (41.1) | 319 (38.4) | 782 (48.5) |  |
| Male | 83 (41.3) | 29 (70.7) | 29 (56.9) | 0 (0.0) | 0 (0.0) | 50 (54.9) | 13 (23.6) | 34 (35.4) | 83 (58.9) | 511 (61.6) | 832 (51.5) |  |
|  |  |  |  |  |  |  |  |  |  |  |  |  |
| **Race**, n (%) |  |  |  |  |  |  |  |  |  |  |  | <.0001^1^ |
| White/Caucasian | 189 (94.0) | 40 (97.6) | 40 (97.6) | 70 (100.0) | 37 (100.0) | 74 (92.5) | 39 (90.7) | 68 (78.2) | 122 (87.8) | 704 (86.2) | 1383 (88.9) |  |
| Asian | 3 (1.5) | 0 (0.0) | 0 (0.0) | 0 (0.0) | 0 (0.0) | 2 (2.5) | 1 (2.3) | 1 (1.1) | 3 (2.2) | 9 (1.1) | 19 (1.2) |  |
| Black/African American | 2 (1.0) | 0 (0.0) | 1 (2.4) | 0 (0.0) | 0 (0.0) | 2 (2.5) | 1 (2.3) | 13 (14.9) | 12 (8.6) | 93 (11.4) | 124 (8.0) |  |
| Other | 7 (3.5) | 1 (2.4) | 0 (0.0) | 0 (0.0) | 0 (0.0) | 2 (2.5) | 2 (4.7) | 3 (3.4) | 2 (1.4) | 4 (0.5) | 21 (1.3) |  |
| American Indian/Alaskan Native | 0 (0.0) | 0 (0.0) | 0 (0.0) | 0 (0.0) | 0 (0.0) | 0 (0.0) | 0 (0.0) | 2 (2.3) | 0 (0.0) | 7 (0.9) | 9 (0.6) |  |
| Missing | 0 | 0 | 10 | 0 | 1 | 11 | 12 | 9 | 2 | 13 | 58 |  |
|  |  |  |  |  |  |  |  |  |  |  |  |  |
| **Education**, n (%) |  |  |  |  |  |  |  |  |  |  |  | <.0001^1^ |
| < High School | 4 (2.3) | 1 (2.6) | 3 (7.1) | 1 (1.6) | 0 (0.0) | 7 (9.6) | 2 (6.1) | 9 (11.7) | 12 (9.5) | 190 (24.5) | 229 (16.0) |  |
| High School Grade/GED | 47 (26.7) | 10 (26.3) | 13 (31.0) | 21 (34.4) | 10 (32.3) | 29 (39.7) | 10 (30.3) | 20 (26.0) | 40 (31.7) | 256 (33.1) | 456 (31.9) |  |
| Some College/Vocational School | 72 (40.9) | 19 (50.0) | 18 (42.9) | 31 (50.8) | 14 (45.2) | 25 (34.2) | 14 (42.4) | 35 (45.5) | 52 (41.3) | 182 (23.5) | 462 (32.3) |  |
| College/Post Graduate Degree | 53 (30.1) | 8 (21.1) | 8 (19.0) | 8 (13.1) | 7 (22.6) | 12 (16.4) | 7 (21.2) | 13 (16.9) | 22 (17.5) | 146 (18.9) | 284 (19.8) |  |
| Missing | 25 | 3 | 9 | 9 | 7 | 18 | 22 | 19 | 15 | 56 | 183 |  |
|  |  |  |  |  |  |  |  |  |  |  |  |  |
| **Married**, n (%) |  |  |  |  |  |  |  |  |  |  |  | <.0001^1^ |
| Married/Marriage like relationship | 95 (81.9) | 31 (75.6) | 22 (78.6) | 41 (67.2) | 20 (83.3) | 67 (85.9) | 29 (67.4) | 42 (70.0) | 65 (46.1) | 503 (61.8) | 915 (65.1) |  |
| Single/Divorced/Widowed | 21 (18.1) | 10 (24.4) | 6 (21.4) | 20 (32.8) | 4 (16.7) | 11 (14.1) | 14 (32.6) | 18 (30.0) | 76 (53.9) | 311 (38.2) | 491 (34.9) |  |
| Missing | 85 | 0 | 23 | 9 | 14 | 13 | 12 | 36 | 0 | 16 | 208 |  |
|  |  |  |  |  |  |  |  |  |  |  |  |  |
| **Total number of medications: Mean (SD)** | N/A | 7.1 (3.59) | 6.2 (3.23) | 3.4 (2.79) | 4.3 (3.97) | 9.6 (4.48) | 5.1 (4.53) | N/A | 8.5 (4.12) | 7.9 (4.44) | 7.5 (4.48) | <.0001^2^ |
| 0 to 4 |  | 10 (24.4) | 13 (25.5) | 48 (68.6) | 25 (67.6) | 9 (9.9) | 31 (58.5) |  | 16 (11.4) | 158 (20.2) | 310 (24.5) | <.0001^1^ |
| 5 to 9 |  | 20 (48.8) | 33 (64.7) | 19 (27.1) | 9 (24.3) | 42 (46.2) | 13 (24.5) |  | 76 (54.3) | 386 (49.4) | 598 (47.3) |  |
| 10 to 14 |  | 11 (26.8) | 5 (9.8) | 3 (4.3) | 3 (8.1) | 40 (44.0) | 9 (17.0) |  | 48 (34.3) | 238 (30.4) | 357 (28.2) |  |
| Missing |  | 0 | 0 | 0 | 1 | 0 | 2 |  | 1 | 48 | 349 |  |
|  |  |  |  |  |  |  |  |  |  |  |  |  |
| **General Health**, n (%) | N/A | N/A | N/A | N/A | N/A | N/A | N/A | N/A |  |  |  | <.01^1^ |
| Excellent/Very good |  |  |  |  |  |  |  |  | 34 (27.0) | 273 (34.7) | 307 (33.7) |  |
| Good |  |  |  |  |  |  |  |  | 74 (58.7) | 334 (42.5) | 408 (44.7) |  |
| Fair/Poor |  |  |  |  |  |  |  |  | 18 (14.3) | 179 (22.8) | 197 (21.6) |  |
| Missing |  |  |  |  |  |  |  |  | 15 | 44 | 702 |  |
|  |  |  |  |  |  |  |  |  |  |  |  |  |
| **Literacy: Confidence in filling forms**, n (%) | N/A | N/A | N/A | N/A | N/A | N/A | N/A |  | N/A |  |  | 0.32^1^ |
| Not at all/little bit |  |  |  |  |  |  |  | 12 (13.2) |  | 65 (8.1) | 77 (8.6) |  |
| Somewhat |  |  |  |  |  |  |  | 14 (15.4) |  | 105 (13.1) | 119 (13.4) |  |
| Quite a bit |  |  |  |  |  |  |  | 27 (29.7) |  | 241 (30.1) | 268 (30.1) |  |
| Extremely |  |  |  |  |  |  |  | 38 (41.8) |  | 389 (48.6) | 427 (47.9) |  |
| Missing |  |  |  |  |  |  |  | 5 |  | 30 | 723 |  |
| N/A, Not applicable  ^1^Chi-Square p-value; ^2^Kruskal-Wallis p-value | | | | | | | | | | | | |

Table 2. OPTION^12^ scores per study.

|  | Name of Study | | | | | | | | | |  | |
| --- | --- | --- | --- | --- | --- | --- | --- | --- | --- | --- | --- | --- |
|  | Chest Pain (N=201) | DAD (N=41) | Diabetes (N=51) | Osteo I (N=70) | Osteo II (N=38) | Statin Choice (N=91) | Graves Disease (N=55) | IADAPT (N=96) | TRICEP (N=141) | SDM4Afib (N=830) | Total (N=1614) | P-value |
| **OPTION^12^** |  |  |  |  |  |  |  |  |  |  |  | <.0001^1^ |
| Mean (SD) | 16.8 (12.09) | 38.4 (25.85) | 40.6 (18.92) | 39.0 (21.84) | 47.7 (14.05) | 9.6 (12.00) | 35.2 (9.27) | 40.8 (16.24) | 22.1 (10.26) | 31.1 (12.15) | 29.2 (16.03) |  |
| Median | 16.7 | 43.8 | 35.4 | 32.8 | 46.9 | 4.2 | 35.4 | 42.2 | 21.9 | 31.3 | 29.2 |  |
| Range | 0.0, 60.4 | 0.0, 83.3 | 12.5, 83.3 | 2.1, 89.6 | 17.7, 83.3 | 0.0, 54.2 | 14.6, 56.3 | 4.2, 70.8 | 0.0, 50.0 | 0.0, 64.6 | 0.0, 89.6 |  |
| ^1^Kruskal-Wallis p-value | | | | | | | | | | | | |


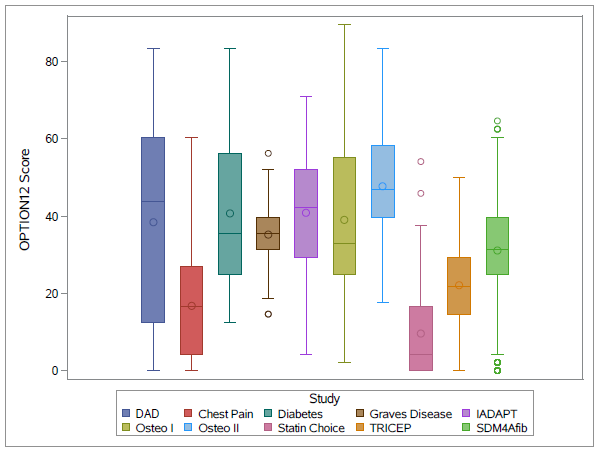


Figure 1. OPTION^12^ scores per study.
