# Supplementary material for: Patient Characteristics and the Extent to Which Clinicians Involve Patients in Decision Making: Secondary Analyses of Pooled Data
Source: Med Decis Making. 2024 Mar 4;44(3):346–56. doi: 10.1177/0272989X241231721 (PMC10988989; doi:10.1177/0272989X241231721)
Supplement: sj-docx-2-mdm-10.1177_0272989X241231721 – Supplemental material for Patient Characteristics and the Extent to Which Clinicians Involve Patients in Decision Making: Secondary Analyses of Pooled Data [file sj-docx-2-mdm-10.1177_0272989X241231721.docx]

**Appendix B.** Multivariable model with interaction effect

Table 3. Multivariable model findings including interaction effect.

| Characteristic | OPTION12-scores  Least Square Mean (95% CI) | *P*-Value |
| --- | --- | --- |
| **Age**:  <55 | 31.3 (23.3, 39.4) | 0.20 |
| 55-64 | 31.0 (23.1, 39.0) |  |
| 65-74 | 31.5 (23.5, 39.5) |  |
| 75+ | 33.3 (25.2, 41.3) |  |
| **Gender**:   Female | 32.5 (22.8, 42.2) | 0.14 |
| Male | 31.0 (21.3, 40.8) |  |
| **Race**:  White | 33.0 (23.4, 42.6) | 0.12 |
| Other | 30.6 (20.6, 40.6) |  |
| **Education**:   < Highschool | 29.9 (21.8,38.0) | 0.02 |
| Highschool/GED | 32.2 (24.3, 40.2) |  |
| Some College/Vocational | 31.0 (23.1, 39.0) |  |
| College/Post-Graduate | 34.0 (26.0, 42.1) |  |
| **Marital Status**:   Married/  Marriage like relationship | 31.0 (22.1, 40.0) | 0.11 |
| Single/Divorced/separated/Widowed | 32.5 (23.6, 41.5) |  |
| **Total Medications**:   0-4 | 32.4 (24.2, 40.7) | 0.51 |
| 5-9 | 31.8 (23.7, 39.9) |  |
| 10+ | 31.1 (22.9, 39.3) |  |
| **Intervention Arm**:   Usual care | 27.6 (18.6, 36.6) | <.0001 |
| SDM conversation tool | 36.0 (27.0, 44.9) |  |
| **Interaction between Age*Arm** |  | 0.01 |
| Usual care, < 55 | 26.7 (18.0, 35.3) |  |
| Usual care, 55-64 | 24.6 (16.3, 32.9) |  |
| Usual care, 65-74 | 28.3 (20.0, 36.5) |  |
| Usual care, 75+ | 30.9 (22.6, 39.2) |  |
| SDM conversation tool, < 55 | 36.0 (27.6, 44.4) |  |
| SDM conversation tool, 55-64 | 37.5 (29.3, 45.8) |  |
| SDM conversation tool, 65-74 | 34.7 (26.5, 43.0) |  |
| SDM conversation tool, 75+ | 35.6 (27.3, 44.0) |  |

** interaction between age categories and arm was found to be significant

*P*= 0.001

Interaction Model: Likelihood Ratio = 8769.9, AIC=8805.9

Base model without interaction: Likelihood Ratio = 8784.7, AIC = 8818.7


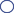

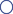

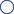

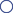

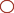

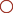

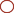

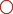

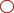

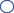


**Fit for OPTION12**

With 95% Confidence Limits

40

30

20

<55

55-64

65-74

75+

Age

Arm Care as usual SDM conversation tool

Figure 2. Graph of interaction effect between age and OPTION^12^ scores.
