# Supplementary material for: Patient Characteristics and the Extent to Which Clinicians Involve Patients in Decision Making: Secondary Analyses of Pooled Data
Source: Med Decis Making. 2024 Mar 4;44(3):346–56. doi: 10.1177/0272989X241231721 (PMC10988989; doi:10.1177/0272989X241231721)
Supplement: sj-docx-3-mdm-10.1177_0272989X241231721 – Supplemental material for Patient Characteristics and the Extent to Which Clinicians Involve Patients in Decision Making: Secondary Analyses of Pooled Data [file sj-docx-3-mdm-10.1177_0272989X241231721.docx]

**Appendix C.** Sensitivity analyses.

Table 4. Univariable model findings of all studies excluding SDM4AFIB (N=784)

| Patient characteristic | OPTION^12^ score  Least Square Mean (95% CI) | *P*-Value |
| --- | --- | --- |
| **Age (years)**  <55 | 33.5 (24.9, 42.1) | 0.31 |
| 55-64 | 31.1 (22.5, 39.7) |  |
| 65-74 | 31.3 (22.6, 39.9) |  |
| >75 | 32.7 (23.6, 41.7) |  |
| **Gender**  Female | 32.2 (22.3, 42.2) | 0.88 |
| Male | 32.0 (22.0, 42.1) |  |
| **Race (**missing: n=45**)**  White | 32.8 (23.0, 42.6) | 0.02 |
| BIPOC | 26.4 (15.6, 39.7) |  |
| **Education** (missing: n=127)  < Highschool | 28.2 (18.6, 37.7) | 0.25 |
| Highschool/GED | 31.6 (23.2, 40.0) |  |
| Some College/Vocational | 32.9 (24.5, 40.0) |  |
| College/Post-Graduate | 33.4 (24.8, 41.9) |  |
| **Marital Status** (missing: n=192)  Married/ Marriage like   relationship | 32.8 (23.4, 42.2) | 0.31 |
| Other (Single, Divorced,   Separated, Widowed) | 31.2 (21.5, 40.9) |  |
| **Total Medications (**missing: n=301**)**  0-4 | 33.4 (23.6, 41.6) | 0.34 |
| 5-9 | 33.7 (23.9, 43.4) |  |
| >10 | 30.9 (20.9, 41.0) |  |
| **Intervention Arm**:   Usual care | 23.8 (14.1, 33.7) | <0.001 |
| SDM conversation tool | 39.5 (29.7, 49.2) |  |

*Note: General Health and Health literacy could not be included in the model as they were not consistently measured across the included studies.*

Table 5. Multivariable Model Findings of all studies excluding SDM4AFIB (N=784)

| Patient characteristic | OPTION^12^ score  Least Square Mean (95% CI) | *P*-Value |
| --- | --- | --- |
| **Age (years)**  <55 | 30.1 (20.0, 40.2) | 0.96 |
| 55-64 | 29.6 (19.6, 39.6) |  |
| 65-74 | 30.1 (20.1, 40.2) |  |
| >75 | 31.0 (20.5, 41.4) |  |
| **Gender**  Female | 30.5 (17.6, 43.4) | 0.74 |
| Male | 29.9 (16.7, 43.1) |  |
| **Race (**missing: n=45**)**  White | 33.3 (20.9, 45.7) | 0.11 |
| BIPOC | 27.1 (12.6, 41.6) |  |
| **Education** (missing: n=127)  < Highschool | 31.6 (20.1, 43.1) | 0.34 |
| Highschool/GED | 27.7 (17.6, 37.8) |  |
| Some College/Vocational | 29.9 (19.9, 39.8) |  |
| College/Post-Graduate | 31.7 (21.4, 41.9) |  |
| **Marital Status** (missing: n=192)  Married/ Marriage like   relationship | 30.8 (19.4, 42.2) | 0.47 |
| Other (Single, Divorced,   Separated, Widowed) | 29.6 (18.1, 41.2) |  |
| **Total Medications (**missing: n=301**)**  0-4 | 30.7 (20.3, 41.1) | 0.94 |
| 5-9 | 30.0 (19.8, 40.5) |  |
| >10 | 29.9 (19.4, 40.5) |  |
| **Intervention Arm**:   Usual care | 22.5 (11.0, 34.0) | <0.0001 |
| SDM conversation tool | 37.9 (26.5, 49.4) |  |
